# 

**Published:** 1855-03

**Authors:** 


VOL. XXXI.] [NO. 183.

THE

### WESTERN JOURNAL

OF

### MEDICINE AND SURGERY.

NEW SERIES. MARCH, 1855. VOL. II—NO. 3.

EDITED BY

LUNSFORD P. YANDELL, M. D.

PROFESSOR OF PHYSIOLOGY AND PATHOLOGICAL ANATOMY, IN THE UNIVERSITY OF LOUISVILLE.

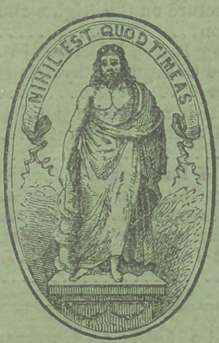

LOUISVILLE, KY:

PRINTED AND PUBLISHED BY J. F. BRENNAN,

*Cor. of Market & First Streets.*

☞PRICE, $3 A YEAR—INVARIABLY IN ADVANCE.—POSTAGE, 2 CENTS A NUMBER.☜

